# THE NEED TO APPEAR HEALTHY: CONCEALMENT OF CHRONIC ILLNESS, PRIVACY, AND SELF-SUFFICIENCY AMONG OLDER NIGERIANS

**DOI:** 10.1093/geroni/igae098.1717

**Published:** 2024-12-31

**Authors:** Kafayat Mahmoud, Tamara Baker, Darlingtina Esiaka, Saliu Balogun

**Affiliations:** Boston University, Boston, Massachusetts, United States; University of North Carolina, Chapel Hill, Chapel Hill, North Carolina, United States; University of Kentucky College of Medicine, University of Kentucky, Kentucky, United States; University of Tasmania, Hobart, Tasmania, Australia

## Abstract

Prior research has highlighted the beneficial impact of social networks and social support on older adults’ physical and psychosocial wellbeing. The impact of the relationship between chronic illness and social networks on the psychosocial wellbeing of older Nigerians remains understudied. This study explored how older Nigerians with chronic illnesses navigate the physical, mental, and emotional changes due to their chronic disease diagnosis within their social contexts. The current qualitative study used semi-structured in-depth interviews with 19 purposively sampled older adults, aged 50 years plus, chronically ill, and receiving clinical care to examine the role of social networks in how chronically ill older Nigerians cope with their diagnosis. Three main themes reflecting participants’ experiences emerged from the findings: 1) closely-knit circles, 2) privacy and self-sufficiency, and 3) body image. Results show that chronically ill older Nigerians prefer to keep the knowledge of their conditions strictly within their close family circles. It was considered horrific to inform friends, community members, and religious groups about one’s chronic illness. Findings further reveal that the need to appear healthy to one’s social network stems from the fear of being discriminated against and attempts to maintain some level of normalcy when interacting with others. Additionally, feelings of inferiority and shame limited their participation in social activities and social network maintenance. We discuss the implication of the results for the mental wellbeing and quality of life of chronically ill older Nigerians and make recommendations for policies and resources that can improve the wellbeing of chronically ill Nigerians.

